# Transcriptome-Wide Study Revealed That N6-Methyladenosine Participates in Regulation Meat Production in Goats

**DOI:** 10.3390/foods12061159

**Published:** 2023-03-09

**Authors:** Juhong Zou, Yujian Shen, Jianwei Zou, Jingsu Yu, Yuhang Jiang, Yanna Huang, Qinyang Jiang

**Affiliations:** College of Animal Science and Technology, Guangxi University, 100 East University Road, Nanning 530004, China

**Keywords:** Duan goats, Nubian goats, M6A, omics analysis, meat production, muscle development

## Abstract

In mammals, skeletal muscle development is a complex biological process regulated by many factors. N6-methyladenosine (m6A) RNA modification plays an important role in many biological processes. However, the regulation of m6A on skeletal muscle growth and development in adult goats remains unclear. In this study, Duan goats (DA) and Nubia goats (NBY), both female and 12 months old, were selected as the research objects, and m6A-Seq and RNA-Seq were mainly used to detect the difference of m6A modification and gene expression during the development of the *longissimus dorsi* (LD) muscle in the two breeds. The results showed that compared with DA, the meat production performance of NBY was better than that of DA, and the modification level of m6A was higher than that of DA in LD. The m6A-Seq of LD indicated m6A peaks were mainly enriched in the coding sequence (CDS) and stop codon. A total of 161 differentially methylated genes (DMGs) and 1294 differentially expressed genes (DEGs) were identified in two breeds. GO and KEGG analysis showed that DMGs were closely related to cellular metabolism, and most of DMGs were enriched in pathways related to energy metabolism, muscle growth and development, mainly MAPK signaling pathway, Wnt signaling pathway and CGMP-PKG signaling pathway. The DEGs were significantly enriched in actin binding, calcium ion binding, angiogenesis, and other biological processes, and most of them were enriched in PI3K-Akt and CGMP-PKG signaling pathways. Combined analysis of m6A-Seq and RNA-Seq data revealed a negative correlation between differentially methylated m6A levels and mRNA abundance, and mRNA expression of the gene with m6A peak near 3′UTR will decrease. In addition, 11 DMGs regulating cell differentiation, muscle growth and development were identified. This study displayed the m6A profiles and distribution patterns in the goat transcriptome, determined the potential role of m6A modification in muscle growth and provided a new reference for the further study of goat skeletal muscle development.

## 1. Introduction

RNA modifications are a form of regulation at the post-transcriptional level and play an important regulatory role in cellular function and gene expression [1]. To date, more than one hundred different RNA modifications have been identified in eukaryotes [2]. The internal modifications of mRNA mainly include N6-methyladenosine modification (m6A), N1-methyladenosine modification (m1A), 5-Methylcytosine modification (m5C), etc. The N6-methylladenosine (m6A) is the most common methylation modification in mRNA [3,4,5,6], which is involved in almost all aspects of RNA metabolism and mediates a variety of life processes [7,8,9,10], widely present in various eukaryotes, such as mammals, plants, Drosophila and yeast. Similar to DNA and histone methylation, m6A methylation is dynamic and reversible. M6A occurs through the methyltransferase complex (writers), which consists mainly of METTL3, METTL14, WTAP and other components [11], and then is removed by the demethylases (erasers) FTO and ALKBH5 [12,13]. M6A binding proteins (readers) recognize m6A modified bases [14,15], thereby activating downstream regulatory pathways and mediating mRNA splicing, export, translation and degradation [9,10,16,17,18,19,20].

The m6A modification is indispensable in regulating gene expression and participating in various bioprocesses, including the regulation of skeletal muscle development in animals [21]. The skeletal muscle tissue in adult animals accounts for about 40–60% of body weight [22], and it is the main source of meat products for livestock. The growth rate of skeletal muscle determines the meat production, and the meat production determines the livestock production [23,24]. In recent years, with the deeper understanding of RNA methylation and the development of high-throughput sequencing technology, the regulatory role of mRNA m6A methylation in animal skeletal muscle and muscle cell development has been gradually revealed. Tao X et al. used m6A sequencing to identify that m6A-modified genes of wild boar—Landrace and Rongchang pigs—were breed specific in *longissimus dorsi* (LD), and exerted different physiological functions in different breeds [25]. Dang Y et al. found by m6A-Seq that m6A modification was involved in the growth and development of beef cattle LD tissue, and the differentially expressed m6A genes were mainly involved in skeletal muscle contraction, steroid biosynthesis process and REDOX process [26]. Petrosino JM et al. discovered that the skeletal muscle size of mice during hypertrophy was related to the increase in METTL3 and m6A levels [27]. In goat longissimus muscle, differential gene GADD45B screened by m6A-Seq can activate p38 MAPK pathway to drive muscle differentiation [28]. In summary, m6A modification plays an important regulatory role in skeletal muscle. However, so far, m6A has been studied relatively little in large livestock goats, and its distribution within the transcriptome of adult goat skeletal muscle has not been reported, which greatly hinders the exploration of m6A mechanisms in goat skeletal muscle.

Duan goats (DA), produced in Duan Yao Autonomous County, is one of the local excellent breeds raised in Guangxi of China. Raising DA has always been the main source of economic income for the Duan people. DA’s coat color is all white, all black or hemp color, and the meat is delicate but grows slowly and does not produce much meat. Meat production trait, as an important economic trait affecting production efficiency, has been a research hotspot in the field of goats genetic breeding. The meat production performance of goats is mainly reflected by carcass weight, net meat weight, dressing percentage and other indicators [29,30]. The weight of adult DA is about 20–40 kg, with a dressing percentage of 40–44% and a net meat percentage of 29–34% [31]. In contrast, Nubian goats (NBY), which were introduced to China from the Nubian region of Africa, grow much faster. Adult NBY have a higher pre-slaughter body weight, dressing percentage and net meat percentage than DA. The phenotypes of the two breeds are significantly different. At present, the level of m6A modification and the distribution of m6A transcriptome range in the skeletal muscle of DA and NBY is not clear, nor is it clear whether m6A is involved in regulating meat production in goats.

Here, we obtained the first transcriptome-wide m6A methylation profile in DA and NBY by m6A-Seq and elucidated the features of m6A modifications in the skeletal muscle of adult goats. We analyzed the regulatory differences of m6A modifications in the two breeds, and identified some differential m6A genes that regulate muscle differentiation and were involved in skeletal muscle growth and development. This work will help to understand the effects of m6A modification for skeletal muscle in goats, and provide a new reference for exploring the role of m6A in skeletal muscle and the mechanism of meat production in goats.

## 2. Materials and Methods

### 2.1. Animals

Five Duan female goats (9.28 ± 0.62 kg body weight) were from Duan Breeding Conservation Farm in Guangxi, and five Nubian female goats (12.65 ± 1.05 kg body weight) were from Green World Core breeding Farm in Guangxi. The goats were 2 months old at the start of the experiment, kept for 10 months in the teaching practice base of College of Animal Science and Technology of Guangxi University and slaughtered at 12 months of age. Each goat was kept in a pen measuring 3.0 m by 1.5 m, where they can walk freely. Bamboo pads (30 cm thick in summer and 40 cm in winter) were laid inside the pen to keep warm. The average temperature of the pens was maintained at 20–27 °C and the average humidity was 55–70%. The goats received free basic daily rations and water. The composition and nutrient content of the basal diet of goats were shown in Table S1. Goat pens were cleaned and disinfected regularly to keep them ventilated, hygienic and dry.

### 2.2. Sample Collection and Ethics Statement

Ten goats were slaughtered in the large animal slaughtering room at the Animal Science Teaching and Experimental base of Guangxi University when they reached the predetermined slaughter age. After 24 h of prohibition of eating, they were stunned by electrocution and killed by bloodletting through their carotid arteries. The LD of the left side of each carcass at the tenth–thirteenth rib was rapidly separated and collected into cryopreservation tubes, which were immediately frozen in liquid nitrogen (−196 °C). After 1 h, the obtained LD samples were brought back to the laboratory, immediately transferred to the −80 °C refrigerator for freezing and taken out after 3 days for experiment. We would randomly select three LD tissues from each group in DA and NBY, a total of six samples, which will be used for m6A-Seq, WB and LC-MS/MS. Sample collection and experiments were conducted following the guidelines for the care and use of laboratory animals formulated by the Ministry of Agriculture of China. All procedures have been approved by the Animal Protection and Utilization Committee of Guangxi University and the Animal Experimental Ethics Committee (approval number: GXU-2022-118).

### 2.3. Determination of Meat Production Performance of Goats

The main indexes of goat meat production performance are as follows: pre-slaughter body weight, carcass weight, net meat weight, carcass bone weight, dressing percentage, net meat percentage and meat-to-bone ratio. The carcass weight, bone weight and net meat weight of ten goats were recorded after slaughter, and the dressing percentage, net meat percentage and meat-to-bone ratio of each goat were calculated. The dressing percentage refers to the percentage of carcass weight to pre-slaughter body weight, which is calculated as dressing percentage (%) = (carcass weight/pre-slaughter body weight) × 100. The net meat percentage refers to net meat weight as a percentage of carcass weight, i.e., (%) = (net meat weight/carcass weight) × 100. Meat–bone ratio is the ratio of net meat weight to carcass bone weight, i.e., = net meat weight/carcass bone weight.

### 2.4. Western Blotting

LD muscles were ground into a powder with liquid nitrogen, and protein lysate RIPA (Solarbio, Beijing, China) was added for re-suspension, which was lysed on ice for 30 min and centrifuged at 13,000 rpm at 4 °C for 10 min, and the supernatant was collected into a new 1.5 mL Centrifuge tube. A total of 2 µL was taken out to determine the protein content in the supernatant according to the protein concentration determination kit (Beyotime, Beijing, China), and the reagent was prepared according to the instructions. A 1× loading buffer was added to extracted protein samples, and after boiling in a water bath for 10 min, the denatured protein samples were added to gel swimlanes, respectively. After running at 110 V for about 1.5 h, the colloids were taken out and placed into a wet membrane transfer system. After running at 200 mA at 4 °C for 1 h, the proteins were transferred to PVDF membranes (Bio-rad, Hercules, CA, USA). The membrane was placed in a sealing solution for 2 h at room temperature and cleaned with TBST. The dilution ratio of the primary antibody was 1:1500, corresponding to protein primary antibodies, such as anti-GAPDH, anti-METTL3, anti-METTL14, anti-WTAP, anti-FTO and anti-ALKBH5 (Abclone, Wuhan, China), which were added and incubated at 4 °C overnight, then rinsed with 1× TBST 3 times, 10 min each. Secondary antibody (Abclonal, Wuhan, China) was incubated at 37 °C for 1 h with a dilution ratio of 1:5000 and rinsed with 1× TBST 3 times, 10 min each. After covering the PVDF membrane with ECL luminescent solution (CWbio, Beijing, China), the imager (Bio-rad, Hercules, CA, USA) was exposed and photographed. The gray level of protein bands was analyzed by Image J software(Version : v1.8.0).

### 2.5. LC-MS/MS Quantification of m6A Levels

Total RNA was isolated using TRIzol reagent (Invitrogen, Waltham, MA, USA) following the manufacturer’s instruction. A total of 1 µg of total RNA was digested by 4 µL nuclease P1 in 40 µL buffer solution at 37 °C for 12 h, followed by incubating with 1 µL alkaline phosphatase at 37 °C for 2 h. RNA solution was diluted to 100 µL and injected into LC-MS/MS. The nucleosides were separated by reverse-phase high-performance liquid chromatography on an Agilent C18 column, coupled with mass spectrometry detection using AB SCIEX QTRAP 5500. Pure nucleosides were used to generate standard curves, from which the concentrations of adenosine (A) and m6A in the sample were calculated. The level of m6A was then calculated as a percentage of total unmodified A.

### 2.6. High-Throughput m6A & mRNA Sequencing

Total RNA was isolated and purified using TRIzol reagent (Invitrogen, Carlsbad, CA, USA) following the manufacturer’s procedure. The RNA amount and purity of each sample were quantified using NanoDrop ND-1000 (NanoDrop, Wilmington, DE, USA). The RNA integrity was assessed by Bioanalyzer 2100 (Agilent, Santa Clara, CA, USA), and confirmed by electrophoresis with denaturing agarose gel. Poly (A) RNA was purified from 50 μg total RNA using Dynabeads Oligo (dT) using two rounds of purification. Then, the cleaved RNA fragments were incubated for 2 h at 4 °C with m6A-specific antibody (No. 202003, Synaptic Systems, Göttingen, Germany) in IP buffer (50 mM Tris-HCl, 750 mM NaCl and 0.5% Igepal CA-630). Finally, ethanol precipitation was used. Then, the IP RNA was reverse-transcribed to create the cDNA by SuperScript™ II Reverse Transcriptase (Invitrogen, cat. 1896649, USA), which were next used to synthesize U-labeled second-stranded DNAs with E. coli DNA polymerase I (NEB, cat.m0209, Ipswich, MA, USA), RNase H (NEB, cat.m0297, USA) and dUTP Solution (Thermo Fisher, cat. R0133, Waltham, MA, USA). An A-base is then added to the blunt ends of each strand, preparing them for ligation to the indexed adapters. Each adapter contains a T-base overhang for ligating the adapter to the A-tailed fragmented DNA. At last, we performed the 2 × 150 bp paired-end sequencing (PE150) for IP RNA and input RNA on an Illumina Novaseq™ 6000 (LC-Bio Technology C, Ltd., Hangzhou, China) following the vendor’s recommended protocol.

### 2.7. Bioinformatics Analysis of m6A-Seq and RNA-Seq Data

We used fastp (https://github.com/OpenGene/fastp, accessed on 16 July 2022) software to perform quality control on the raw data of IP samples and Input samples, including removing joints, repetitive sequences, and low-quality sequences to obtain Clean Data. Clean Data were compared to a reference genome (Latin: Capra hircus, genome version: v101) through HISAT2 (http://daehwankimlab.github.io/hisat2, accessed on 16 July 2022), and the file format was bam. The bamfile used the R package exomePeak (https://bioconductor.org/packages/exomePeak, accessed on 20 July 2022) for peak calling analysis and genetic difference peak analysis. The obtained tdf or bigwig format files can be used with the IGV software (http://www.igv.org, accessed on 22 July 2022) for visual display. ChIPseeker (https://bioconductor.org/packages/ChIPseeker, accessed on 25 July 2022) was used to comment on the peak. MEME2 (http://meme-suite.org, accessed on 10 August 2022) and HOMER Simpson (http://homer.ucsd.edu/homer/motif, accessed on 15 August 2022) were used for the analysis of motif. The differentially expressed mRNA was identified by *p*-value and folding change. The gene assembly and quantification software was Stringtie (https://ccb. jhu. edu//software/stringtie, accessed on 2 September 2022), the quantification method was FPKM (total exon fragments/mapped reads (millions) × exon length (kB)), and the R package edgeR was used (Https://bioconductor.org/packages/edgeR, accessed on 4 September 2022) for difference analysis. Finally, gene GO function enrichment analysis and KEGG pathway analysis was performed by KOBAS3.0 (http://kobas.cbi.pku.edu.cn/index.php, accessed on 20 September 2022).

### 2.8. Statistical Analysis

The data were expressed as mean ± standard deviation (SD) and statistically analyzed using a one-way analysis of variance (ANOVA) and a least significant difference (LSD) test using SPSS 22.0 software. The *p* < 0.05 means significant difference, and *p* < 0.01 or *p* < 0.001 indicates extremely significant difference.

## 3. Results

### 3.1. Comparative Analysis of Meat Production Performance between DA and NBY

The results of production index determination showed that compared with DA, the pre-slaughter body weight, carcass weight, net meat weight and carcass bone weight of NBY were significantly higher (*p* < 0.01), and the dressing percentage and net meat percentage of NBY were also higher than those of DA (*p* < 0.05) (Table 1). The above results indicated that NBY has better meat production performance than DA under the same feeding and management conditions.

### 3.2. Analyzed m6A Modification Levels of Skeletal Muscle in DA and NBY

**Figure 1:**
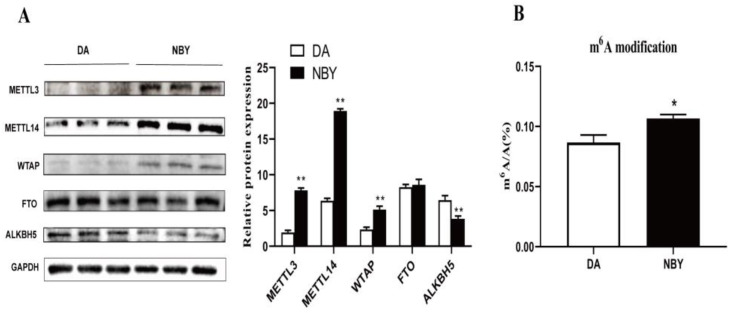
Protein expression of m6A-related enzymes in DA and NBY LD and detection of global m6A levels. (**A**) The expression of METTL3, METTL14, WTAP, FTO and ALKBH5 protein abundance was detected by Western blot (** *p* < 0.01). (**B**) M6A/A ratio of DA and NBY samples were measured by LC-MS/MS. The bar shows the mean from n = 3 sample replicates (* *p* < 0.05).

Protein expression of m6A modification related enzymes in LD muscle, as shown in Figure 1A. The expression levels of m6A methyltransferases METTL3, METTL14 and WTAP in NBY were significantly higher than those in DA (*p* < 0.05). The expression level of m6A demethylase ALKBH5 in NBY was significantly lower than that in DA (*p* < 0.05). There was no significant difference in FTO expression between the two breeds. To elucidate the level of m6A modification of LD muscle in DA and NBY, liquid chromatography-tandem mass spectrometry (LC-MS/MS) was used to detect m6A modifications (Figure 1B). We compared the m6A/A ratio and found that the ratio was in the range of 0.08%–0.11% in goats, and the ratio of NBY was higher than DA (*p* < 0.05). These results suggested that the m6A modification level of LD muscle in NBY was higher than that in DA, and may be involved in regulating muscle development.

### 3.3. Transcriptome-Wide Detection of m6A Modification in DA and NBY

From m6A-Seq, more than 75,400,000 reads were obtained from each NBY sample, and more than 71,800,000 reads were obtained from each DA sample (Table S2). After filtering out low-quality data, the comparison software HISAT2 [32] was used to compare the preprocessed valid data with reference genomes of Capra hircus (Genome version: v101). More than 63,300,000 valid reads from each NBY sample were mapped to the reference genome, and similarly, more than 63,200,000 valid reads from each DA sample were mapped to the reference genome (Table S2). More than 69% of the IP reads in LD were uniquely mapped to the reference genome (Table S1). From RNA-Seq, 6.9–7.3 million raw reads were obtained from NBY samples, more than 5.7 million valid reads were mapped to the reference genome, 7.1–7.5 million raw reads were obtained from DA samples and more than 6.3 million valid reads were mapped to the reference genome (Table S2). According to the regional information of the reference genome, mapped reads were located between exons, introns and intergenes. In general, exonic regions have the highest percentage of mapped reads, while intronic and intergenic regions are usually lower.

**Figure 2:**
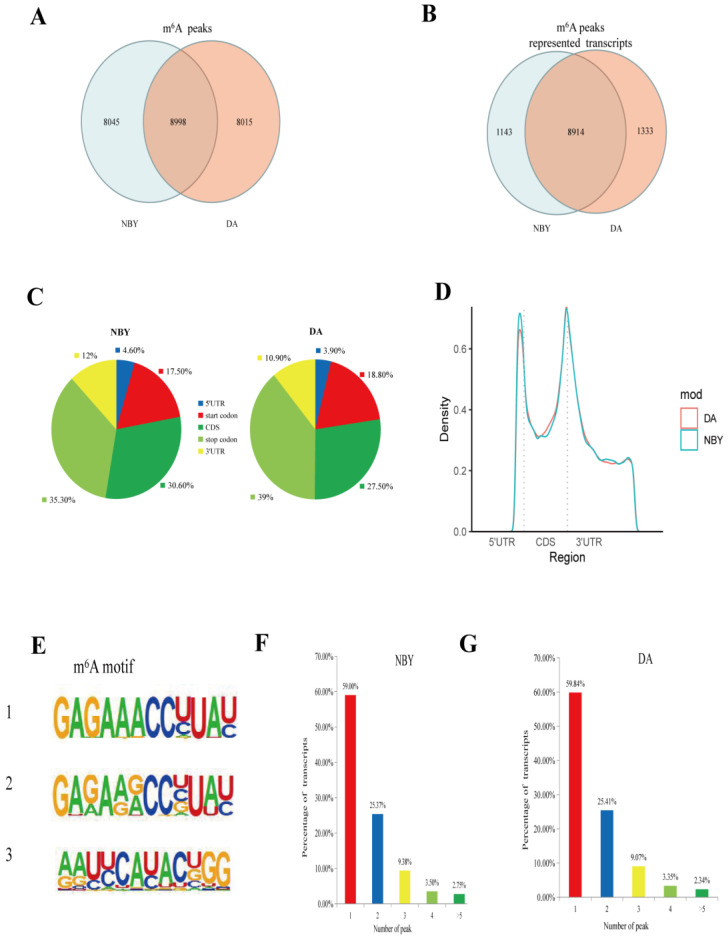
Transcriptome-wide m6A sequencing and m6A peaks analysis of NBY and DA. (**A**) Common and unique m6A peaks in NBY and DA. (**B**) Venn diagram of m6A peaks-represented transcripts of two groups. (**C**) The proportion of m6A peaks distribution in the indicated regions in NBY and DA. (**D**) Enrichment of m6A peaks along with transcripts in NBY and DA. Each transcript was divided into three parts: 5′UTR, CDS, and 3′UTR. (**E**) The top three motifs enriched across m6A peaks identified from NBY and DA. (**F**) The distribution of m6A-modified peaks per gene in NBY m6A genes. (**G**) The distribution of m6A-modified peaks per gene in DA m6A genes.

Genome-wide m6A peak scanning was performed using the R package exomePeak [33]. After calculating the *p*-value of the candidate peak region, the default *p*-value is <0.05, and the region less than the *p*-value is considered to be a peak. A total of 17,013 m6A peaks were detected in DA, representing transcripts of 10,247 genes, and 17,043 m6A peaks were detected in NBY, representing transcripts of 10,057 genes (Table S1). Among them, there were 8015 unique peaks in DA and 8045 unique peaks in NBY, representing transcripts of 1333 genes and 1143 genes, respectively (Table S3; Figure 2A,B). Importantly, in the NBY vs. DA, we detected 164 differential methylation peaks, representing transcripts of 161 genes (Table S4).

To verify the preferential location of m6A in transcripts, we next studied the distribution of m6A peaks in the entire transcriptome of DA and NBY. The m6A peaks were assigned to five non-overlapping transcriptional intervals, which were 5′UTR, start codon, CDS, stop codon and 3′UTR, and counted the distribution of m6A peaks in each segment. About 27.5%–30.6% of m6A peaks fell in the CDS, 35.3%–39% in the stop codon, 17.5%–18.8% in the start codon, 10.9%–12% in the 3′UTR, and very few m6A peaks were distributed in the 5′UTR area, with more than two-thirds of the m6A peaks falling near the CDS and the stop codon region. (Table S3; Figure 2C). There was no significant difference regarding the distribution of m6A peaks in the LD muscle between two breeds. Analysis of the relative positions of m6A peaks showed that these peaks were mainly located near the stop codon or the beginning of 3′-UTR (Figure 2D). We searched for motifs enriched in the area around the m6A peaks to determine whether the identified m6A peaks contained RRACH motifs (where R stands for adenine or guanine, A stands for m6A, and H stands for non-guanine bases), and the results showed that the motifs do exist (Figure 2E). In addition, the number of m6A peaks were different in each m6A modified mRNA. As shown in Figure 2F,G, the number of m6A peaks varies from 1 to 16 in a single gene, while nearly 80% of methylated transcripts contained only one or two m6A peaks. Only 2% of genes had more than five m6A peaks in their transcripts. The number distribution of m6A peaks was very similar in DA and NBY transcripts.

### 3.4. Biologic Pathways Associated with m6A-Modified Genes

**Figure 3:**
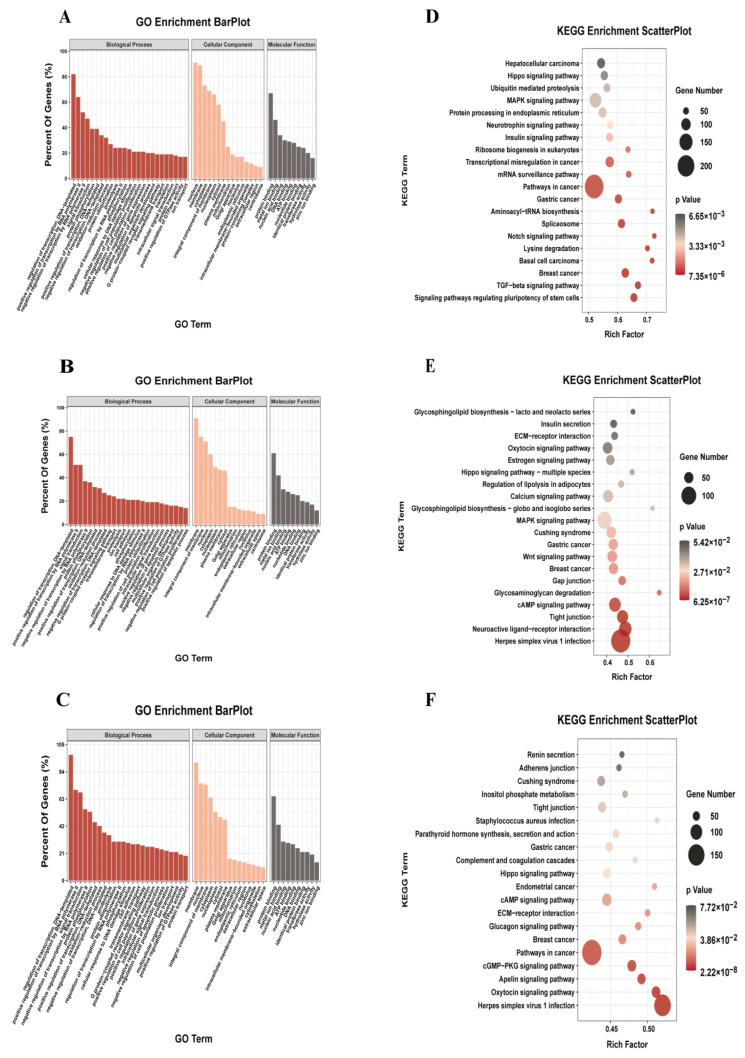
Functional analysis of genes associated with m6A peaks. (**A**) GO analysis of common genes associated with m6A peaks in NBY and DA. (**B**) Gene ontology analysis of unique genes associated with m6A peaks in NBY. (**C**) Gene ontology analysis of unique genes associated with m6A peaks in DA. (**D**) KEGG analysis of common genes associated with m6A peaks in NBY and DA. (**E**) KEGG analysis of unique genes associated with m6A peaks in NBY. (**F**) KEGG analysis of unique genes associated with m6A peaks in DA.

As shown in Figure 3B, in the DA and NBY, there were 8914 common m6A genes (whose transcripts carry m6A peaks, referred to as the m6A gene), as well as 1143 and 1333 unique m6A genes. To further determine the regulation role of m6A modification in muscle growth and analyze the functional pathway of m6A gene in LD development, we conducted GO enrichment analysis and KEGG pathway analysis for the m6A genes. GO enrichment analysis results showed that common m6A genes were mainly involved in protein phosphorylation, oxidation-reduction process, positive regulation of cell population proliferation and positive regulation of gene expression (Table S5; Figure 3A). The unique m6A genes of NBY were associated with regulation of transcription, positive regulation of cell population proliferation, positive regulation of gene expression and multicellular organism development (Table S6; Figure 3B). However, the unique m6A genes of DA were associated with the protein ubiquitination, intracellular signal transduction and positive regulation of GTPase activity (Table S7; Figure 3C). KEGG pathway analysis results showed that common m6A genes were mainly enriched in MAPK signaling pathways, insulin signaling pathway, Notch signaling pathway and TGF−beta signaling pathway (Table S8; Figure 3D). The unique m6A genes of NBY were mainly enriched in the calcium signaling pathway, Wnt signaling pathway and cAMP signaling pathway (Table S9; Figure 3E), which were related to muscle growth development and energy metabolism. However, the unique m6A genes of DA were mainly enriched inositol phosphate metabolism, tight junction and glucagon signaling pathway (Table S10; Figure 3F). These results indicated that m6A modification had a regulatory function for muscle growth and development in goat.

**Figure 4:**
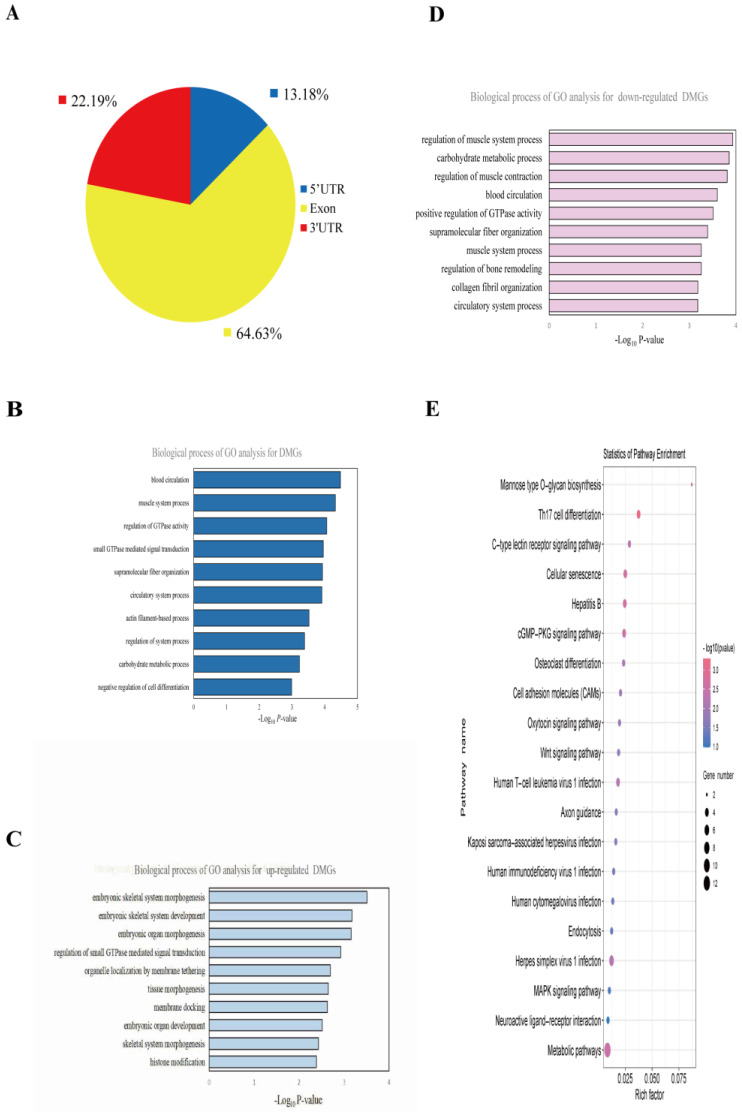
Distribution and functional enrichment analysis of DMGs. (**A**) Distribution of differential methylation peaks. (**B**) GO analysis of differentially methylated genes (DMGs). (**C**) The top ten significantly enriched pathways for the up-regulated DMGs. (**D**) The top ten significantly enriched pathways for the down-regulated DMGs. (**E**) KEGG analysis of DMGs.

In the NBY vs. DA, more than 60% of the differential methylation peaks occurred in the exons of the transcripts (Figure 4A). A total of 164 differential methylation peaks were screened, and 161 differentially methylated genes (DMGs) were identified. Among them, 55 differential methylation peaks were up-regulated, which was associated with the expression of 54 DMGs, and 109 differential methylation peaks were down-regulated, which was associated with the expression of 107 DMGs. GO biological processes analysis results showed that total DMGs were mainly involved in blood circulation, muscle system process and regulation of GTPase activity (Table S11; Figure 4B). The up-regulated DMGs were related to embryonic skeletal system morphogenesis, embryonic skeletal system development and embryonic organ morphogenesis (Table S12; Figure 4C). The down-regulated DMGs were related to the regulation of muscle system process, carbohydrate metabolic process, regulation of muscle contraction and blood circulation (Table S13; Figure 4D). Moreover, KEGG pathway analysis showed that total DMGs were enriched mainly in the Wnt signaling pathway, cGMP-PKG signaling pathway and MAPK signaling pathway (Table S14; Figure 4E). These results suggested that DMGs play an important role in regulating muscle development in goats.

### 3.5. Identification of Differentially Expressed Genes (DEGs) by RNA-Seq

**Figure 5:**
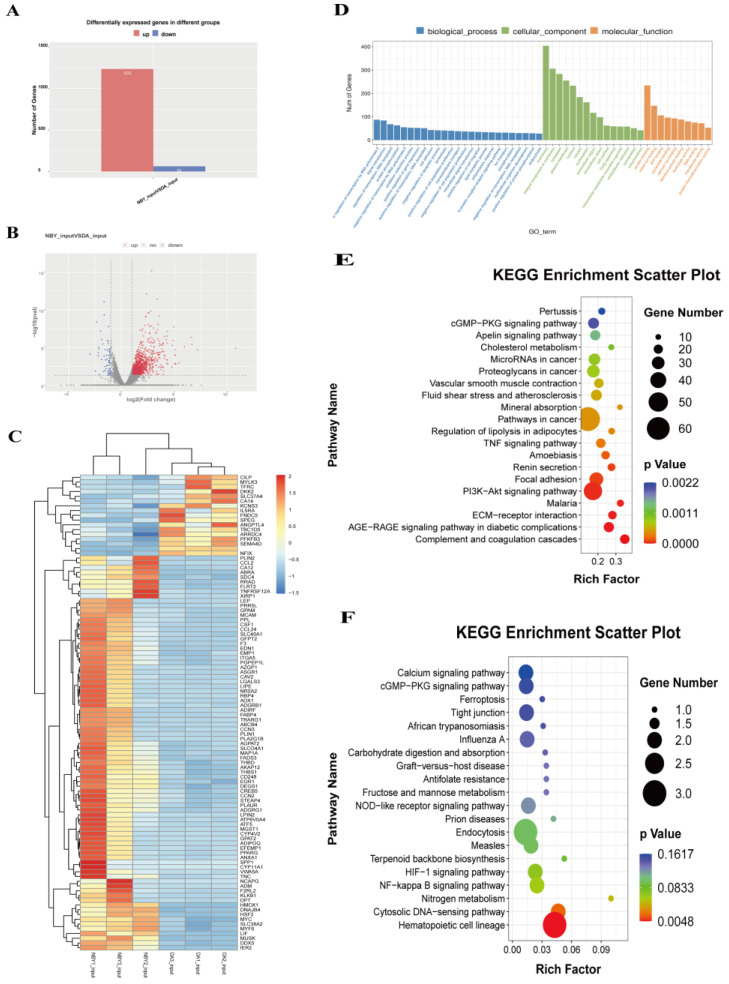
Analysis of differentially expressed genes (DEGs) between NBY and DA. (**A**) Number of up-regulated and down-regulated DEGs. The red column indicates up-regulated DEGs and the blue column indicates down-regulated DEGs. (**B**) The volcano of DEGs. (**C**) Heat map of DEGs. (**D**) GO analysis for DEGs. (**E**) KEGG pathway analysis of up-regulated DEGs. (**F**) KEGG pathway analysis of down-regulated DEGs.

RNA-Seq was used to elucidate the mRNA expression difference between DA and NBY LD tissues and to map the associated biological pathways. Compared to DA, 1294 DEGs were found in NBY, of which 1232 were up-regulated and 62 were down-regulated (Table S15; Figure 5A–C). GO analysis showed that DEGs were significantly enriched in biological processes including extracellular space, actin-binding, calcium ion binding and angiogenesis (Table S16; Figure 5D). These bioprocesses played important biological roles in the proliferation and differentiation of myoblasts, energy metabolism in muscle cells and regulation of myogenesis-related life activities. The KEGG enrichment analysis diagrams and data of up-regulated and down-regulated DEGs were shown in Tables S17 and S18 and Figure 5E,F. It was worth mentioning that the up-regulated DEGs in NBY were significantly enriched in the PI3K-Akt and CGMP-PKG signaling pathway, and the two pathways were essential for the growth and development of muscles. These results suggested that DEGs may play a key role in the development of skeletal muscle.

### 3.6. A Conjoint Analysis of m6A-Seq and RNA-Seq Data in DA and NBY

**Figure 6:**
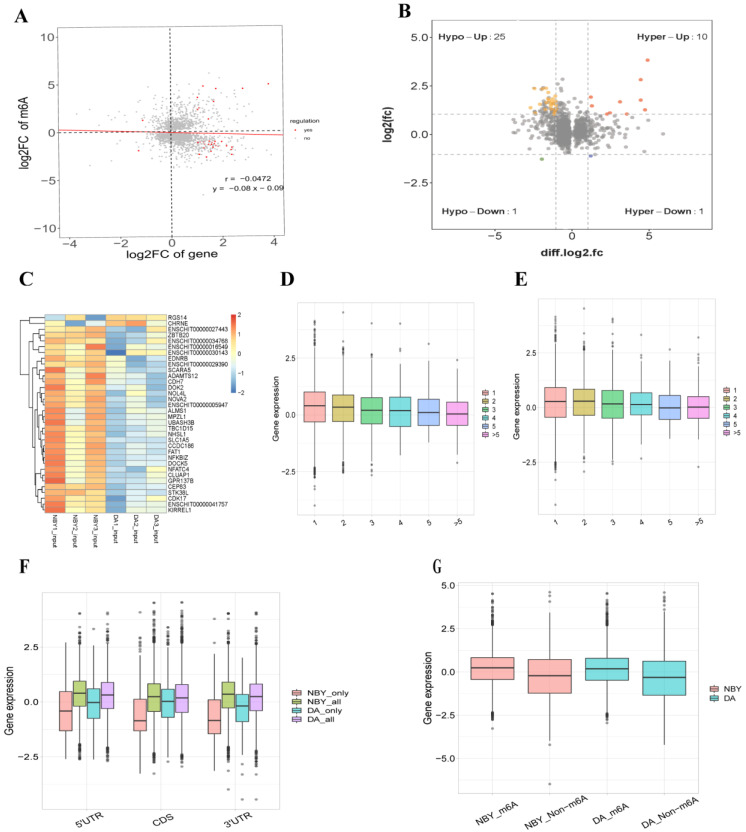
Conjoint analysis m6A-Seq and RNA-Seq data in DA and NBY. (**A**) Negative correlation of differentially methylated m6A peaks and gene expression level (r = −0.0472; y = −0.08x − 0.09). (**B**) Distribution of genes with a significant change in both differentially methylated m6A peaks and mRNA expression in DA and NBY. (**C**) Cluster heat maps of 34 genes with significant differences in m6A peaks and RNA level. (**D**) Expression levels of genes carrying the different number of m6A peaks in NBY. (**E**) Expression levels of genes carrying the different number of m6A peaks in DA. (**F**) The m6A modification sites and gene expression in all m6A peaks, NBY-unique peaks and DA-unique peaks. (**G**) Overall expression level of m6A methylated and non-m6A methylated genes in DA and NBY.

Cross-analysis of m6A-Seq and RNA-Seq data showed a negative correlation of differential methylation m6A peaks and genes expression abundance in DA and NBY (Figure 6A). Compared with DA, 34 genes showed significant difference in both m6A peaks and RNA level in NBY (fold change > 2, *p* < 0.05) (Table S19; Figure 6B,C). We classified these genes into four types: hypermethylated and upregulated genes were called hyper-up, and there were ten; hypermethylated and downregulated genes were called hyper-down, and there was one; hypomethylated and downregulated genes were called hypo-down, and there was one; the genes that were hypomethylated and upregulated were called “hypo-up”, and there were 25 of them. Furthermore, among the 34 genes, 11 genes (TBC1D15, DOK2, NFATC4, MTPN, FAT1, GOLGB1, DOCK5, CLUAP1, SLC1A5, CHRNE and ALMS1) were found to be closely related to muscle growth and development.

To understand whether the number of m6A peaks was related to gene expression levels, the relationship between the m6A peaks and gene expression level in NBY and DA was studied. Obviously, the results showed that different genes had different amounts of m6A peaks. By analyzing the relative expression levels of these genes, we found that the expression level of genes with more than three m6A peaks were much lower than the expression level of genes with one or two m6A peaks (Figure 6D,E). Furthermore, the relationship between the distribution position of m6A peaks along with the mRNA transcript and gene expression level were also studied. According to the modification position of m6A in RNA, the RNA transcripts were divided into 5′UTR, CDS and 3′UTR, and the m6A peaks were divided into all m6A peaks, NBY-unique peaks and DA-unique peaks. As a result, compared with 5′UTR or CDS m6A modification, RNA transcripts with m6A modification around 3′UTR tend to have reduced gene expression levels (Figure 6F). In terms of overall gene expression levels, the expression level of m6A genes were lower than that of non-m6A genes in two breeds (Figure 6G).

## 4. Discussion

In this study, we mainly performed transcriptome-wide analysis of DA and NBY skeletal muscle tissues by m6A-Seq, constructed m6A modification profile of goat skeletal muscle, and determined that m6A modification was involved in the regulation of muscle growth and development in goats. In addition, we confirmed that DMGs present in DA and NBY muscle tissues are enriched in pathways and bioprocesses associated with muscle development.

The dressing percentage and net meat percentage can reflect the meat production performance of the animals. The dressing percentage and net meat percentage of NBY were significantly higher than those of DA, indicating that NBY have faster growth and development of muscle tissue and higher meat production. Earlier studies have shown that the increase in meat production in animals was inseparable from skeletal muscle hypertrophy, and the process of skeletal muscle hypertrophy was accompanied by up-regulation of m6A methyltransferase expression and elevated abundance of m6A modification [27]. As such, we detected the expression levels of methyltransferases METTL3, METTL14 and demethylase ALKBH5 in DA and NBY by WB. WB results showed that the protein levels of METTL3 and METTL14 were higher in NBY than in DA, whereas the level of ALKBH5 was lower. High expression of METTL3/14 and low expression of ALKBH5 may affect the abundance of m6A modifications in skeletal muscle [34]. LC-MS/MS is one of the scientific methods for detecting the overall modification level of m6A [35]. We detected the overall level of m6A in LD muscle by LC-MS/MS and found that the level of m6A modification were higher in NBY than that in DA. NBY had higher m6A modification level and meat production, indicating that m6A modification level in goat skeletal muscle may be positively correlated with muscle hypertrophy and meat production. We speculate that DA belong to the local breed in Guangxi, and the breeding of selection is low, with muscle growth and development being slow [31]. NBY are an imported breed with a high breeding of selection, and muscle growth and development is rapid [36]. Therefore, for NBY, more genes related to muscle growth and more regulatory mechanisms are needed during development; m6A is eukaryotic epigenetic modification, which may play a regulatory role in muscle growth and development [28].

The level of m6A modification usually has a constant range in the organism, and its drastic changes can affect the normal physiological processes of the organism. In the model organism Arabidopsis thaliana and mice, the content of mRNA m6A in Arabidopsis thaliana leaves was about 0.4%–0.8% [37], and in mouse spermatozoa it was between 0.3% and 0.8% [38]. In this work, the m6A modification level of NBY and DA LD muscle was detected to be 0.08%–0.11%, which was lower than that of Arabidopsis thaliana and mice. This difference may be caused by different detection methods, different species or different tissue samples. The mRNA m6A sites of the goat were mainly enriched around stop codon, CDS and 3’UTR. Their distribution was similar to that previously reported in humans and mice [8]. By counting the base arrangement of the m6A peak region, we found that m6A modification was more likely to occur on the conservative sequence GGACU, and the m6A methylation process of other mammalian mRNAs also required this motif [37,38]. The m6A modification profiles of NBY and DA were very similar, and their modification characteristics were highly consistent with those of human beings, rats, pigs and other eukaryotes reported previously, indicating that m6A was highly conservative in most species.

GO analysis explored the unique m6A methylation genes of two breeds. NBY-unique m6A methylation genes, regulated by m6A modification, were involved in transcriptional regulation, cell proliferation and positive regulation of gene expression; these processes were associated with muscle growth and development. However, DA-unique m6A methylation genes, which were involved in intracellular signaling and protein ubiquitination, were less enriched in muscle-related bioprocesses. The unique m6A modification genes were involved in the regulation of different pathways and physiological processes in the two breeds, which may determine their different muscle phenotype. Most importantly, KEGG pathway analysis revealed that 161 DMGs were mainly enriched in signaling pathways closely related to muscle growth and energy metabolism, such as the Wnt signaling pathway [39], cGMP-PKG signaling pathway [40] and MAPK signaling pathway [41]. Previous studies have proved that Wnt signaling pathway plays a key role in maintaining myogenic differentiation and promoting myogenesis in human and mouse data [42,43]. In the canonical Wnt signaling pathway, myogenic cells lacking *β*-catenin exhibited delayed differentiation, whereas myoblasts with constitutively active *β*-catenin underwent precocious growth arrest and differentiation, and a precise level of *β*-catenin activity was essential for regulating the amplification and differentiation of muscle stem cell descendants during adult myogenesis [44]. In addition, the activation of hypoxia-inducible factor (HIF)-1α under hypoxia, in murine skeletal myoblasts, led to the activation of MyoD through the non-classical Wnt/*β*-catenin pathway, promoting myotubular hypertrophy [45]. Furthermore, the overexpression of Wnt5a and circSNX29 activated the non-classical Wnt5a/Ca^2+^ pathway, facilitated myogenic cell differentiation and inhibited cell proliferation [46]. The cGMP-PKG signaling pathway was involved in the modulation of glucose uptake in skeletal muscle [47] and the stimulating effects of cGMP on glucose transport required the activation of PKG in muscle cells [48]. As one of the most important pathways of energy metabolism, MAPK signaling pathway plays a crucial role in in the formation, regeneration, movement and injury repair of skeletal muscle [49,50]. To summarize the above findings, we concluded that activating the Wnt, cGMP-PKG and MAPK signaling pathways through DMGs may perform a key function during the development and differentiation of goat muscle tissues.

In the work, association analysis showed that m6A modification abundance was negatively correlated with gene expression levels, which was consistent with two previous studies [51,52]. We also found that the expression of genes containing more than three m6A sites was much lower than that of genes containing one or two m6A sites, suggesting that m6A can regulate the degradation of mRNA [53]. Importantly, among the 34 genes with significant differences in m6A peak and RNA level screened by association analysis, 12 genes related to muscle growth and development were the focus of our attention. TBC1D15, a highly conserved protein belonging to TBC (Tre-2/Bub2/Cdc16), functions as a GTPase-activating protein (GAP) for Rab proteins and affects glucose uptake in skeletal muscle by regulating the glucose transporter protein GLUT4 [54]. DOK2 is a key regulatory gene of the insulin signaling axis and glucose metabolism in skeletal muscle [55]. NFATc4, a member from the Nuclear Factor of Activated T cells (NFATs) transcription factor family, plays a pivotal role in the development of cardiac hypertrophy. NFATc4 is dephosphorylated by calcineurin and translocated from the cytoplasm to the nucleus to regulate the expression of hypertrophic genes [56]. MTPN is a myotrophin factor. During the process of skeletal muscle hypertrophy, myotrophin promotes myotube hypertrophy and increases myotube diameters while inhibiting myoblast proliferation [57]. FAT1 cadherin, as a regulator of muscle morphogenesis, is required in the myogenic lineage to control the polarity of progenitor migration [58]. GOLGB1 is associated with growth and carcass traits of animals, and the 65-bp indel in GOLGB1 can be used as a molecular marker to improve breeding efficiency [59]. DOCK5 (cytokinin 5) is required for myoblast fusion during embryogenesis in vertebrates [60]. For CLUAP1, CRISPR/cas9-mediated CLUAP1 knockout revealed a new phenotype that affects the actin cytoskeleton [61]. Uptake of macrophage-derived glutamine by satellite cells through glutamine transporter SLC1A5 (solute vector family 1 member 5) activates mTOR and promotes proliferation and differentiation of satellite cells [62]. CHRNE is one of the most common genes responsible for congenital myasthenic syndromes, and mutations can lead to muscle hypotonia, weakness or developmental delays [63]. ALMS1 is associated with myocyte differentiation and cell cycle control [64]. These genes have different regulatory effects and are associated with muscle growth and development, indicating the potential mechanism of functional genes regulating goat skeletal muscle development.

## 5. Conclusions

In conclusion, this study demonstrated the complete transcriptome map of goat skeletal muscle and revealed the m6A transcriptome distribution characteristics of goat muscle. Differential m6A modification level and m6A modified genes were closely related to the growth and development of muscle tissue between the two breeds. The methylation level of m6A was correlated with the meat production of goat. The results presented here provide a reference for further research on the mechanism of meat production of skeletal muscle in goats.

## Figures and Tables

**Table 1:** Meat production performance indices of DA and NBY.

Trait Index	DA (n = 5)	NBY (n = 5)
Pre-slaughter body weight (kg)	38.54 ± 3.20 ^B^	66.65 ± 9.77 ^A^
Carcass weight (kg)	17.25 ± 1.68 ^B^	31.56 ± 4.17 ^A^
Net meat weight (kg)	14.86 ± 1.89 ^B^	29.17 ± 5.18 ^A^
Carcass bone weight (kg)	4.56 ± 0.79 ^B^	8.30 ± 1.11 ^A^
Dressing percentage (%)	51.78 ± 0.01 ^b^	57.45 ± 0.03 ^a^
Net meat percentage (%)	38.49 ± 0.02 ^b^	43.54 ± 0.02 ^a^
Meat-bone ratio	2.78:1	2.74:1

Values are shown as mean ± SD, n = 5. Values within a column followed by different lowercase letters are significantly different (*p* < 0.05). Values within a column followed by different capital letters are significantly different (*p* < 0.01).

## Data Availability

The data that support the findings of this study are available from the corresponding author upon reasonable request.

## Supplementary Materials

The following supporting information can be downloaded at: https://www.mdpi.com/article/10.3390/foods12061159/s1, Tables S1–S3. Summary of basal diets, sequence data and read alignment statistics. Table S4. Differentially methylated genes in NBY vs DA groups. Table S5. Gene ontology analysis of common genes associated with m6A peaks in NBY and DA. Table S6. Gene ontology analysis of unique genes associated with m6A peaks in NBY. Table S7. Gene ontology analysis of unique genes associated with m6A peaks in DA. Table S8. KEGG analysis of common genes associated with m6A peaks in NBY and DA. Table S9. KEGG analysis of unique genes associated with m6A peaks in NBY. Table S10. KEGG analysis of unique genes associated with m6A peaks in DA. Table S11. GO analysis of differentially methylated genes. Table S12. The top ten significantly enriched biological processes of GO analysis for the up-regulated DMGs. Table S13. The top ten significantly enriched biological processes of GO analysis for the down-regulated DMGs. Table S14. KEGG analysis of differentially methylated genes. Table S15. Differetially expressed genes in RNA-Seq. Table S16. Differetially expressed genes in GO analysis. Table S17. KEGG pathway analysis of up-regulated DEGs. Table S18. KEGG pathway analysis of down-regulated DEGs. Table S19. Compared with DA, the genes with significant changes in both m6A level and mRNA transcriptome abundance in NBY.
