# Supplementary material for: Transcriptome-Wide Study Revealed That N6-Methyladenosine Participates in Regulation Meat Production in Goats
Source: Foods. 2023 Mar 9;12(6):1159. doi: 10.3390/foods12061159 (PMC10048064; doi:10.3390/foods12061159)
Supplement: Supplementary file 1 [file foods-12-01159-s001.zip › Table S1-3. Summary of basal diets, sequence data and read alignment statistics.pdf]

Supplementary Table S1. Dietary ingredients and nutrient content of basal diets in goats.

Ingredients	Content(%)	Nutrition levels	Content(%)
Corn	16.80	Crude protein	17.20
Palm Kernel Expeller	20.50	Neutral detergent fiber	43.51
Peanut vine	11.00	Crude fat	2.7
Soybean meal	4.00	Calcium,Ca	0.69
Molasses	2.00	Total Phosphorus,TP	0.43
Manioc waste	12.00	Crude ash	7.76
Bagasse	6.00	Nitrogen free exteact	51.2
Corn skin	4.00	Acid detergent fiber	22.65
Cassava alcohol residue	9.00		
rice mill by-product	5.00		
Urea	1.20		
Dicalcium phosphate	0.40		
Limestone	1.00		
Bentonite	4.00		
Rumen protected fat	1.00		
Premix feed	2.10		
Total	100.00		

Supplementary Table S2 . Summary of sequence data and read alignment statistics

	Sample ID	Raw_Reads	Valid_Reads	Mapped reads	Unique Mappe reads	m6A peaks/ m6A modified genes	Expressed gene
IP-seq	NBY_1	76857182	75311702	63339205(95.06%)	48017455(72.07%)	17043 /10057	
	NBY_2	75517400	73964574	65359243(95.07%)	49368439(71.81%)		
	NBY_3	75484228	74360232	64469901(95.00%)	48736154(71.82%)		
	DA_1	71898700	70467066	63275859(95.03%)	47095979(70.73%)	17013 /10247	
	DA_2	72516484	70945312	63603638(94.94%)	46750353(69.78%)		
	DA_3	74159794	72778100	65084437(95.15%)	48409880(70.77%)		
Input-seq	NBY_1	69999430	69091326	57562200(94.93%)	42342050(69.83%)		14924

Note:NBY_1,NBY_2 and NBY_3 mean the sample 1, sample 2 and sample 3 of muscle tissue from Nubian goats ,respectively. DA_1,DA_2 andDA_3 mean the sample 1, sample 2 and sample 3 of muscle tissue from Duan goats ,respectively.

NBY_2	73469490	72605266	64064223(95.00%)	47337553(70.20%)	13824
NBY_3	73930092	73057100	61472094(94.41%)	45704636(70.20%)	14582
DA_1	75670170	74845360	66609319(95.43%)	49034233(70.25%)	13894
DA_2	75727778	74852736	66223736(94.59%)	48157964(68.79%)	13877
DA_3	71977324	70970374	63274152(95.11%)	45873121(68.95%)	13918

Supplementary Table S3. Unique peaks and common peaks between IP and input.

Tissue	Unique peaks	Represented transcripts	Common peaks	Total peaks					
				Total	5' UTR	Start codon	CDS	Stop codon	3'UTR
NBY	8045	1143	8998	17043	784	2983	5215	6016	2045
DA	8015	1333		17013	663	3198	4678	6620	1854

Note: NBY means the sample of muscle tissue from Nubian goats. DA means the sample of muscle tissue from Duan goats.
